# The moderation of genetic risk for ten major psychiatric and substance use disorders by the genetic aptitude for educational attainment

**DOI:** 10.1038/s41380-025-03022-z

**Published:** 2025-04-17

**Authors:** Kenneth S. Kendler, Henrik Ohlsson, Jan Sundquist, Kristina Sundquist

**Affiliations:** 1 Virginia Institute for Psychiatric and Behavioral Genetics, Virginia Commonwealth University, Richmond, VA USA; 2 Department of Psychiatry, Virginia Commonwealth University, Richmond, VA USA; 3 Center for Primary Health Care Research, Department of Clinical Sciences, Lund University, Malmö, Sweden; 4 University Clinic Primary Care Skåne, Region Skåne, Sweden

**Keywords:** Genetics, Diagnostic markers

## Abstract

We seek to clarify the impact of the Genetic Aptitude for Educational Attainment (GAEA) on risk for 10 psychiatric disorders divided into 4 groups: Internalizing, Externalizing, Eating/Compulsive and Psychotic. Educational attainment and psychiatric and substance use disorder information were obtained from national Swedish registries. GAEA and disorder-specific family genetic risk score (FGRS) were calculated from extended pedigrees. In males, information on IQ and resilience was obtained from the Swedish conscript registry. Affected individuals were born in Sweden from 1973–1995 to Swedish born parents. Controlling for disorder specific FGRS, GAEA were negatively and substantially associated with risk for externalizing and internalizing disorders, minimally associated with psychotic disorder risk and positively and modestly associated with risk for eating/compulsive disorders. While the majority of GAEA effect on risk for externalizing disorders was mediated through impact on IQ, for internalizing disorders, mediation was largely through resilience. For externalizing and internalizing disorders, interactions between GAEA and disorder specific FGRS were robust and negative – the slope of disorder risk with increasing genetic liability was steepest in those with low GAEA. For eating disorders, interactions were modest and positive –the slope of risk with increasing genetic liability being steepest in individuals with high GAEA. We found that the impact of GAEA on risk for psychiatric and substance can be substantial and varies widely across disorders in magnitude, direction, and mediation. GAEA also often interacts, sometimes robustly, with disorder specific genetic risk factors. Comprehensive risk models for psychiatric disorders should consider the inclusion of GAEA.

Educational attainment influences risk for a wide variety of psychiatric and substance use disorders [1–4]. Both twin [5–7] and molecular genetic studies [8, 9] have shown a significant genetic component to educational attainment. Given the pervasive evidence for a substantial role for genetic risk factors in the etiology of psychiatric disorders [10, 11], the importance of clarifying how *Genetic Aptitude for Educational Attainment* (GAEA) and the genetic risks for individual psychiatric syndromes inter-relate in the etiology of psychiatric illness is self-evident. Several studies have begun to examine this question and demonstrated, for example, a genetic correlation between the risk for major depression (MD) and GAEA [12], evidence that low GAEA and genetic risk for MD positively interact in causing MD [9] and, in a prior study by our team, demonstrating that genetic risk for alcohol use disorder (AUD) and drug use disorder (DUD) both interact with GAEA. Consistent with prior evidence that high educational status can reduce the heritability of alcohol consumption [13, 14], we found that individuals with low GAEA were especially sensitive to the pathogenic effect of high genetic risk in AUD and DUD [15].

GAEA is a complex construct that can likely be broken down into several sub-components. In addition to the expected positive correlation with intelligence, high levels of GAEA are associated with resilience-like constructs, including high levels of self-control and low levels of neuroticism [9, 16]. We are unaware of prior effects to examine the degree to which these various constructs mediate the impact of GAEA on risk for psychiatric and substance use disorders, and especially whether the pattern of mediation differs across disorders.

In this study, we therefore systematically examine the relationship between GAEA and the risk for ten representative psychiatric and substance use disorders from the Swedish nation-wide registries. These ten 10 disorders represent four broad subgroups suggested by prior multivariate studies of genetic risk [17, 18]: *Internalizing disorders*: MD and Anxiety Disorders (AD); *Eating/Compulsive Disorders*: Anorexia Nervosa (AN), Bulimia Nervosa (BN), and Obsessive-Compulsive Disorder (OCD); *Externalizing Disorders*: AUD, DUD and ADHD; and *Psychotic Disorders*: Schizophrenia (SZ) and Bipolar Disorder (BD).

We see to answer, for these disorders, the following three questions:

1. What is the effect of GAEA on risk for these disorders on its own and controlling for each disorder’s primary genetic risk?
2. To what extent is the impact of GAEA on disorder risk mediated through its effect on IQ versus resilience and does this pattern differ across disorders? These analyses can only be performed in males because of the availability of these mediators only in the Swedish conscript registry.
3. Do GAEA and disorder-specific genetic liability interact in the prediction of risk for our 10 disorders and if so, is i) the impact of a high genetic disease risk attenuated or augmented in those with low levels of GAEA and ii) are the pattern of interaction different in men versus women?

## Methods

We collected information on individuals from Swedish population-based registers with national coverage linking each person’s unique personal identification number which, to preserve confidentiality, was replaced with a serial number by Statistics Sweden. We secured ethical approval for this study from the Regional Ethical Review Board in Lund and no participant consent was required (No. 2008/409 and later amendments). Our dataset consisted of all individuals born in Sweden from 1973–1995 to Swedish born parents (N = 1,886,190). In the dataset, we included age of first registration for the ten disorders: MD, AD, OCD, AN, BN, AUD, DUD, ADHD, BD and SZ utilizing ICD-8, 9 and 10 codes from primary care, specialist, and hospital registers as well as prescription and criminal registries [see Appendix Tables 1–2]. In the dataset, individual Familial Genetic Risk Scores (FGRSs) for the ten disorders as well as the GAEA were included. The FGRS and GAEA were calculated using identical methods based on 1st–5th degree relatives to the probands with a mean of 40.1 relatives per proband. We use different terms because, while “risk” is an appropriate phrase to describe liability to a disorder, it is not suitable to describe a propensity for high educational attainment, for which we utilized “aptitude.” Briefly (see Appendix Table 3), they are calculated from morbidity risks for disorders (or aptitude for educational attainment) in relatives, controlling for cohabitation effects, and thus arise from phenotypes in extended pedigrees, not from molecular genetic data. They are standardized by year of birth and county of residence into a z-score with mean = 0 and SD = 1. We also used data from the Military Conscription Register, which includes cognitive assessments for nearly all 18–20-year-old men in Sweden during the inclusion period. The Swedish military service conscription examination involves a full medical assessment including cognitive function (IQ) measured by four subtests representing logical, spatial, verbal, and technical abilities, and a resilience score that was designed by the Swedish military to assess the ability to cope with psychologically stressful situations and was scored on a normally distributed 1–9 scale. During the years covered by this study, this examination was required by law. Of men born in Sweden, only those with serious medical conditions or disabilities were excused (~4.2% of individuals). The global IQ score, derived from a summation of the four subtests, was standardized to give a Gaussian distributed score between one and nine. For each year we standardized the IQ score and the resilience score into a Z-score.

To investigate the effect of GAEA on risk for our 10 representative disorders and controlling for each disorder’s primary genetic risk we used Aalen’s linear hazards model [19]. In this hazard model, the effects of GAEA upon baseline hazard is additive. Follow-up time in months was measured from age 17 until time of first registration for the specific disorder, death, emigration, or end of follow-up (31 December 2018). In Model A1 and A2, aside from year of birth and sex, we include FGRS for the disorder (A1) and the GAEA (A2). In model B we include FGRS for the disorder and the GAEA in the same model. The results from these models are presented as the %-unit increase (for 1 SD unit increase in FGRS/GAEA) in cases per 34 years (which is the mean follow-up for all individuals in the sample). In order to investigate interactions in risk prediction of our 10 disorders for GAEA and the disorder specific FGRS, we included an interaction term between the two variables. The interaction is measured on the additive scale as defended previously [20]. For these models we present figures where we predict the rates of the disorder (during 34 years) at different levels of FGRS/GAEA. Statistical analyses were performed using SAS statistical software, version 9.4 [21] and the R-package Timereg in R [22]. To gain insight into the pattern of the mediation effect of GAEA on the disorder risk we used a path model, where the pathways from GAEA were divided into three paths, one direct path, one path through IQ and one path through resilience. The fit function was weighted least squares. Mplus version 7.31 was used for model fitting [23]. The standardized path estimates are presented as the total direct effect, the indirect effect, and effects that are mediated through IQ/Resilience. Note that for this analysis we could only use male individuals from the military conscript register (N = 509,155). Individuals who were registered for the specific disorder prior to age 19 were excluded from the analyses.

## Results

We studied individuals born in Sweden from 1973–1995 to Swedish born parents (N = 1886,190). The mean (SD) year of birth was 1984 (6.7) and the mean (SD) age at follow-up was 33.8 (7.1). The sample was 48.8% female. The lifetime prevalences of our ten disorders are seen in Table 1 and range widely, from 0.20% for SZ to 19.75% for AD.

**Table 1:** Life time prevalences of the disorders considered and the tetrachoric correlation between the FGRS for the Individual disorders and the Genetic Aptitude for Educational Attainment (GAEA).

Disorder	Life-Time Prevalence	Correlation FGRS GAEA (SE)
MD	333,018 (17.66%)	−0.10 (0.00)
AD	372,448 (19.75%)	**−**0.15 (0.00)
OCD	24,491 (1.30%)	**−**0.00 (0.00)
AN	9457 (0.50%)	**−**0.03 (0.00)
BN	5957 (0.32%)	0.01 (0.00)
DUD	106,005 (5.62%)	**−**0.29 (0.00)
AUD	71,554 (3.79%)	**−**0.28 (0.00)
ADHD	62,667 (3.32%)	**−**0.27 (0.00)
BD	27,664 (1.46)	**−**0.04 (0.00)
SZ	3805 (0.20%)	**−**0.01 (0.00)

We also estimate the tetrachoric correlation, in the entire Swedish population, between the FGRS for each disorder and for GAEA. These results suggest three groupings (Table 1). For the externalizing disorders, the genetic correlations were negative and moderate (ranging from −0.27–−0.29). That is, individuals at high genetic risk for these disorders had significantly lower levels of GAEA. The second grouping is formed by the internalizing disorders which also had negative and significant genetic correlations with GAEA but more modest in magnitude (−0.10–−0.15). For the third grouping, the genetic correlation between risk for Eating/Compulsive and Psychotic disorders and GAEA were quite modest and even slightly positive for BN.

We show the prediction of risk for our 10 disorders by the disorder-relevant FGRS and GAEA in univariable and multivariable regression in, respectively, models A and B in Table 2. Four patterns of findings are noteworthy. *First*, for internalizing disorders, FGRS_MD_, FGRS_AD_, and low levels of GAEA all predict increased risk of illness in model A with the disorder specific genetic effects being stronger. In model B, the predictive effect of the GAEA declines much more than the FGRS_MD_ and FGRS_AD,_ consistent with the modest genetic correlation between them. *Second*, the pattern is quite different for our Eating/Compulsive Disorders where high levels of GAEA are significantly associated with risk for all three disorders and, for AN and BN, of greater effect than that seen for FGRS_AN_ and FGRS_BN._ *Third*, for our externalizing disorders, the impact on risk of GAEA is negative and between one-half and one third that seen for the disorder specific risks in the univariable model and declines substantially in the multivariable model likely because of the relatively large correlation between the two genetic risk factors. *Fourth*, the pattern of results differs for our two psychotic disorders. In the univariable results, for SZ, the impact of GAEA on risk is minimal while for BD, it has a main effect of a nearly identical magnitude to that of FGRS_BD_. As expected, given the minimal correlation between these genetic risks and GAEA, the multivariable results are nearly identical to the univariable ones.

**Table 2:** Results from Aalens Linear Hazard models predicting the % increase in the risk for the relevant disorder resulting from one SD increase in the disorder specific FGRS and the Genetic Aptitude for Educational Attainment (GAEA).

		Model A	Model B	Model C
MD	MD FGRS	6.19 (6.12; 6.26)	6.09 (6.02; 6.15)	6.15 (6.09; 6.22)
	GAEA	**−**1.88 (**−**1.94; **−**1.82)	**−**1.42 (**−**1.48; **−**1.36)	**−**1.38 (**−**1.44; **−**1.32)
	Interaction			**−**0.84 (**−**0.90; **−**0.78)
AD	AD FGRS	6.63 (6.56; 6.70)	6.43 (6.36; 6.49)	6.46 (6.39; 6.53)
	GAEA	**−**1.99 (**−**2.05; **−**1.92)	**−**0.81 (**−**0.88; **−**0.73)	**−**1.15 (**−**1.22; **−**1.09)
	Interaction			**−**0.91 (**−**0.98; **−**0.84)
OCD	OCD FGRS	0.38 (0.26; 0.41)	0.38 (0.36; 0.41)	0.39 (0.36; 0.41)
	GAEA	0.08 (0.06; 0.09)	0.08 (0.06; 0.10)	0.08 (0.06; 0.10)
	Interaction			**−**0.02 (**−**0.05; **−**0.00)
AN	AN FGRS	0.09 (0.08; 0.11)	0.09 (0.08; 0.11)	0.09 (0.07; 0.10)
	GAEA	0.13 (0.12; 0.15)	0.13 (0.12; 0.15)	0.14 (0.12; 0.15)
	Interaction			0.02 (0.01; 0.04)
BN	BN FGRS	0.04 (0.03; 0.05)	0.04 (0.03; 0.05)	0.04 (0.03; 0.05)
	GAEA	0.06 (0.06; 0.07)	0.06 (0.05; 0.07)	0.06 (0.05; 0.07)
	Interaction			0.01 (0.00; 0.02)
DUD	DUD FGRS	3.98 (3.91; 4.05)	3.71 (3.64; 3.74)	3.35 (3.29; 3.40)
	GAEA	**−**1.92 (**−**1.96; **−**1.89)	**−**1.15 (**−**1.18; 1.12)	**−**1.25 (**−**1.29; **−**1.22)
	Interaction			**−**0.96 (**−**1.01; **−**0.91)
AUD	AUD FGRS	2.33 (2.29; 2.37)	2.33 (2.29; 2.37)	1.95 (1.91; 1.99)
	GAEA	**−**1.32 (**−**1.35; **−**1.29)	**−**0.82 (**−**0.85; **−**0.80)	**−**0.91 (**−**0.94; **−**0.88)
	Interaction			**−**0.68 (**−**0.71; **−**0.64)
ADHD	ADHD FGRS	2.87 (2.82; 2.92)	2.76 (2.71; 2.82)	2.63 (2.58; 2.68)
	GAEA	**−**1.09 (**−**1.12; **−**1.06)	**−**0.55 (**−**0.58; **−**0.53)	**−**0.57 (**−**0.60; **−**0.54)
	Interaction			**−**0.35 (**−**0.39; **−**0.31)
BD	BD FGRS	0.08 (0.08; 0.09)	0.08 (0.08; 0.09)	0.09 (0.08; 0.09)
	GAEA	**−**0.09 (**−**0.10; **−**0.07)	**−**0.08 (**−**0.10; **−**0.08)	**−**0.08 (**−**0.10; **−**0.06)
	Interaction			**−**0.06 (**−**0.09; **−**0.03)
SZ	SZ FGRS	0.13 (0.12; 0.15)	0.13 (0.12; 0.15)	0.13 (0.12; 0.15)
	GAEA	0.00 (0.00; 0.01)	0.01 (**−**0.01; 0.00)	0.01 (0.01; 0.01)
	Interaction			**−**0.02 (**−**0.03; 0.00)NS

Model A: Univariable models controlled for YoB and sex.

Model B: Multivariable models (FGRS and GAEA are included in the same models) controlled for YoB and sex.

Model C: Multivariable models and the interaction between FGRS and GAEA, controlled for YoB and sex.

We then, in males only, explored, in Table 3, the mediation, through intelligence and resilience, of the impact of GAEA on risk for our 5 most prevalent disorders, where we could obtain stable statistical estimates. The proportion of the GAEA effect mediated by these two variables ranged from 51.6% for AUD to 100% for AD. Of substantial interest, the proportion of the GAEA effect mediated through intelligence was substantially and significantly greater than that mediated through resilience for the two substance use disorders (AUD and DUD) while the opposite result was seen for the two internalizing disorders (AD and MD). The most striking contrast was between AUD where two thirds of the GAEA effects on risk was mediated through intelligence and MD where nine-tenths of the GAEA effect was mediated through resilience.

**Table 3:** Mediation of the Effect of GAEA (with 95% CIs) on Risk for A Selected Set of our Disorders in Males.

							Proportion of Mediation		
	Total Effect of GAEA	Total Direct effect	Total Indirect effect	Indirect via IQ	Indirect via Resilience	Total Proportion Mediated	Through IQ	Through Resilience	*P*-value*
DUD	**−**0.171 (**−**0.177; **−**0.166)	**−**0.079 (**−**0.086; **−**0.072)	**−**0.092 (**−**0.096; **−**0.089)	**−**0.052 (**−**0.055; **−**0.049	**−**0.041 (**−**0.042; **−**0.039)	53.8%	56.5%	44.5%	<0.0001
AUD	**−**0.182 (**−**0.189; **−**0.175)	**−**0.089 (**−**0.097; **−**0.081)	**−**0.094 (**−**0.098; **−**0.090)	**−**0.062 (**−**0.066; **−**0.059)	**−**0.032 (**−**0.033; **−**0.030)	51.6%	66.1%	33.9%	<0.0001
ADHD	**−**0.109 (**−**0.117; **−**0.101)	**−**0.029 (**−**0.038; **−**0.019)	**−**0.080 (**−**0.085; **−**0.075)	**−**0.041 (**−**0.045; **−**0.038)	**−**0.038 (**−**0.040; **−**0.037)	73.4%	51.2%	48.8%	0.127
MD	**−**0.053 (**−**0.058; **−**0.049)	**−**0.014 (**−**0.019; **−**0.008)	**−**0.039 (**−**0.042; **−**0.037)	**−**0.005 (**−**0.007; **−**0.003)	**−**0.035 (**−**0.036; **−**0.033)	73.6%	10.3%	89.7%	<0.0001
AD	**−**0.053 (**−**0.057; **−**0.048)	**−**0.000 (0.005; **−**0.005)	**−**0.053 (**−**0.055; **−**0.050)	**−**0.021 (**−**0.023; **−**0.019)	**−**0.032 (**−**0.033; **−**0.031)	100%	39.6%	60.4%	<0.0001

*Nominal *p* value of test of difference of the proportion of GAEA effect on disorder risk mediated through IQ versus Resilience.

**Fig. 1:**
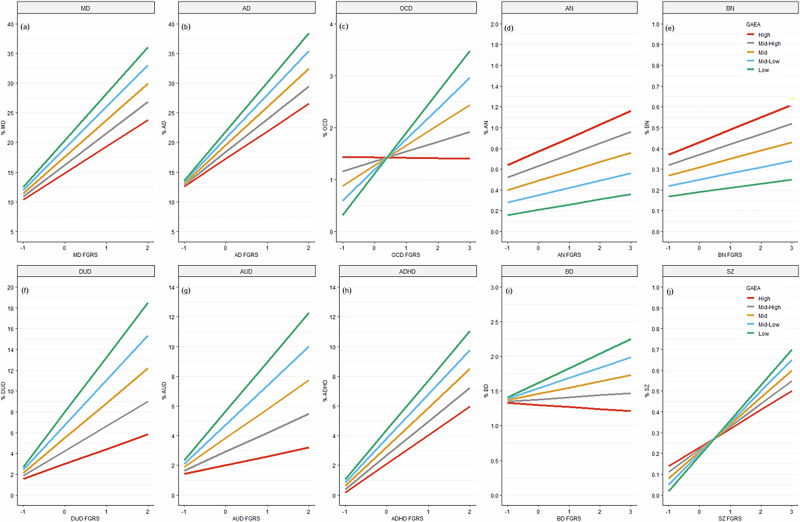
The Impact of GAEA, Disorder Specific Genetic Risk and Their Interaction in the Prediction of Risk for our Ten Disorders in Four Overall Categories. (1) Internalizing Disorders – Major Depression (MD) in (**a**) and Anxiety Disorders (AD) in (**b**); (2) Eating/Compulsive Disorders: Anorexia Nervosa (AN) in (**d**), Bulimia Nervosa (BN), in (**e**) and Obsessive-Compulsive Disorder (OCD) in (**c**); (3) Externalizing Disorders: Alcohol Use Disorder (AUD) in (**g**), Drug Use Disorder (DUD) in (**f**) and ADHD in (**h**); and Psychotic Disorders: Schizophrenia (SZ) in (**j**) and Bipolar Disorder (BD) in (**i**). In each subfigure, the Y axis is the predicted risk for the disorder and the X-axis is the Family Genetic Risk Score (FGRS) for that disorder in standard deviation units. The lines represent the relationship between the level of FGRS for each disorder and its population prevalence as a function of the level of the individuals Genetic Aptitude for Educational Attainment (GAEA) as described by mean Z scores as follows: red line – high 2 SDs above the mean; grey line - mid-high 1 SD above the mean; yellow line – mean; blue line - Mid-low 1 SD below the mean; Green line – low 2 SDs below the mean. Analyses were performed using Aalen’s linear hazards model with interactions defined on an additive scale.

Next, we examine the interaction between disease-specific genetic liability and GAEA on risk for all our disorders both in model C in Table 2 and Fig. 1a–j. We here focus on the figures. Again, we see four major patterns of findings. *First*, for MD, AD, DUD, AUD, and ADHD (Fig. 1a, b, f, g and h), we see a fan-shaped pattern which depicts a negative interaction between the disorder specific genetic liability and GAEA in the prediction of disorder risk. That is, the slope of risk produced by increasing genetic liability is less steep in those with high GAEA and becomes progressively steeper with decreasing levels of GAEA. The spread of the risk – that is the differences in the slope with the highest versus lowest levels of GAEA is greatest for DUD and AUD, intermediate for ADHD and smallest for MD and AD. *Second*, for two disorders – OCD and SZ – (in Fig. 1c and j), we see a cross-over effect. For those with low levels of FGRS_OCD_ and FGRS_SZ_, the rates of illness are highest in those with high levels of GAEA. However, with high disease specific risk, this pattern flips and the highest disorder risk is found in those with the lowest level of GAEA. However, the specific nature of these patterns differ substantially between the two disorders. For SZ, for all levels of GAEA, a strong relationship is seen for increasing disorder risk with increased levels of FGRS_SZ_. But for OCD, for those with the highest level of GAEA, disorder risk is nearly independent of level of FGRS_OCD_. By contrast, for those with the lowest levels of GAEA, risk for the disorder is strongly associated with magnitude of the FGRS_OCD_.

*Third*, for two disorders – AN and BN – (Fig. 1d and e), the risk for disorder is always highest for those with high GAEA and this difference becomes slightly greater as the levels of FGRS_AN_ and FGRS_BN_ increase (albeit, this interaction effect is not statistically significant for BN.) *Fourth*, BD, in Fig. 1i, has a unique result. At quite low genetic risk for BD, GAEA has no impact on disorder risk. As the level of FGRS_BD_ increases, the disorder risk increases for those with low GAES but actually declines slightly with those with high GAEA.

**Fig. 2:**
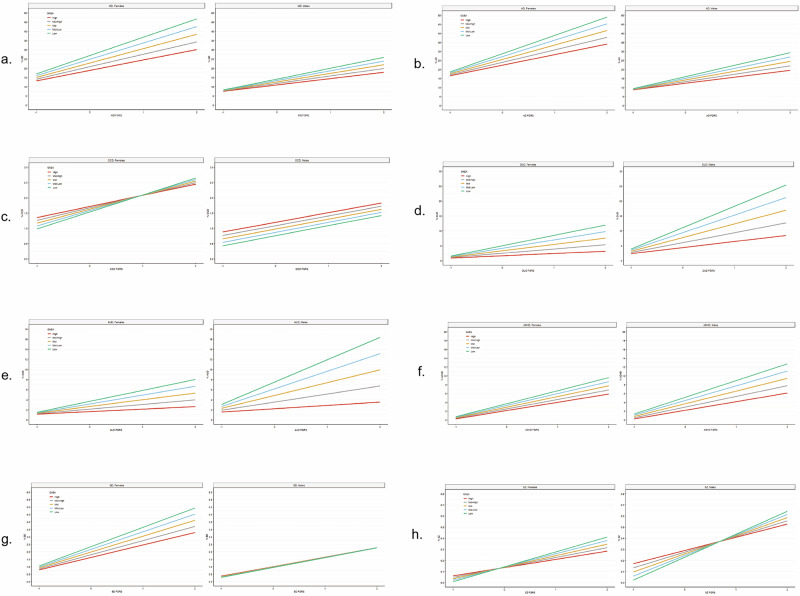
The Impact of GAEA, FGRS for our 8 of our disorders (all but AN and BN) and Their Interaction in the Prediction of Risk for these 10 disorders in Women and Men. These figures are depicted exactly the same way as Fig. 1 with the X axis representing the risk for the specific disorder and the y axis the FGRS for that disorder. The results for females are presented to the left and for males to the right in each sub-figure. The level of GAEA is depicted by colored lines as defined in the legend for Fig. 1. Results are presented for Major Depression (MD) in (**a**), anxiety disorders (AD) in (**b**), obsessive-compulsive disorder (OCD) in (**c**), Drug Use Disorder (DUD) in (**d**), alcohol use disorder (AUD) in (**e**), ADHD in (**f**); bipolar disorder (BD) in (**g**) and schizophrenia (SZ) in (**h**).

Finally, we compared the results of these interactive models in males and females for 8 of our disorders, excluding AN and BN (for details see appendix Table 5). As seen in Fig. 2a–h, the basic shape of the results replicates across the two sexes for 6 of the 8 disorders: MD, AD, DUD, AUD, ADHD, and SZ. In those disorders with substantial sex differences in prevalence, the “spread” in the fan-shaped interactions is more pronounced in the sex with the higher prevalence, i.e., females for MD and AD and males for DUD, AUD, and ADHD. For BD, results in both males and females differ from that seen in the entire population. Females demonstrate a simple fan-shaped interaction while in males, no main effect for GAEA is evident. For OCD, the results in women approximate that seen in the entire population while for males, we see main effects for both GAEA and FGRS_OCD_ but no interaction.

## Discussion

Following up on prior studies of potential interactions between the GAEA and disorder specific genetic liability for psychiatric and substance use disorders [9, 12–15], we find, in what is likely the most comprehensive study to date of this question, a surprisingly complex patterns of findings across our diverse group of ten disorders. Of the many findings presented, we emphasize five general trends. First, in a well powered and representative national Swedish samples, we saw three broad patterns of association between GAEA and genetic risk for our diverse set of psychiatric and substance use disorders. The most robust associations – moderate and negative -- was seen for externalizing disorders, followed by an association that was modest and negative for the internalizing disorder and one that was minimal and in one instance positive for the psychotic and eating disorders. Our nearly zero genetic correlation between GAEA and the FGRS for schizophrenia is broadly consistent with the prior literature which has shown negative, nearly null and slightly positive correlations [24–26]. While most of these findings are consistent with epidemiological studies showing the relationship of familial educational attainment or related variables such as socioeconomic status with risk for these disorders [27, 28], some, particularly, SZ (e.g. [29]), are not. For SZ, this discrepancy could be explained by prior evidence that risk for SZ is robustly predicted by a level of educational attainment below that predicted from family background [30].

Second, we then examined, in Table 1 model A, the impact of GAEA on disorder risk controlling for genetic risk factors for the individual disorder. Of interest, for all three of our Eating/Compulsive Disorders – OCD, AN and BN – the impact of GAEA on risk was, consistent with at least one prior report [31], positive and statistically significant with the estimate being highest for AN. These models again demonstrate the much larger effect on disease risk of GAEA for the externalizing and internalizing compared to the psychotic disorders.

Third, GAEA has been shown to have at least two major components that impact on intelligence and personality-like traits reflecting resilience-like constructs of self-control and low levels of negative emotionality [9, 16]. Using good indices of these constructs from the Swedish conscript registry, we explored mediational pathways for the impact of GAEA on risk for five of our more common disorders. The proportion of the GAEA effect on these disorders ranged from 50–55% for AUD and DUD to 100% for AD. While the majority of mediational effects for DUD, AUD and ADHD were via IQ, the majority of mediation for AD and especially MD was via resilience. These results suggest that the pathways from GAEA to risk for psychiatric and substance use disorders may qualitatively differ in important ways which has implications for preventative measures and understanding of underlying neural mechanisms.

Fourth, we assessed (in Table 1 model C and Fig. 1), the main effect and interactions between GAEA and the disorder specific FGRS for each of our 10 disorders. The interactions were statistically significant for all disorders except SZ (which had the smallest sample size). The interaction effects were diverse and, for many disorders, substantial. Only for the EDs were the interactions qualitatively modest. Across the large majority of the disorders examined, we could reject, with substantial confidence, *the hypothesis that the disorder specific genetic risk and the genetic aptitude for educational attainment act independently of one another on disease risk*. All the externalizing and internalizing disorders displayed classical fan-shaped interactions. That is, both disease specific genetic liability and GAEA likely contributed positively to disease risk, but the impact of disease risk become progressively more important the lower the level of GAEA. Cross-over effects were seen for both OCD and SZ. Although interaction effects were weak for the two EDs, in both AN and BN, disorder specific genetic liability had a modestly strong effect on disease risk at high levels of GAEA. As discussed below, however, caution is necessary in interpreting our associations as being entirely or even largely causal in nature.

Finally, we examined sex differences for 8 of our 10 disorders (male prevalence for EDs was far too low to be meaningful analyzed), the results of which served as a “check” on the stability of our solutions. Reassuringly, the basic shape of the interaction was stable across 6 of these disorders. However, for BD and OCD, both relatively low prevalence conditions, the models differed qualitatively across sexes. We cannot confidently determine whether these are valid sex differences or statistical instabilities of our models.

In interpreting our findings, we would suggest that readers focus on the overall pattern of effects that are likely to be more reliable, such as evidence that genetic risks for psychiatric and substance use disorders often interact with GAEA and GAEA’s effect on individual disorders likely differ in their mediational pathways. Some of our more idiosyncratic findings such as the instability of our results for BD and OCD across sexes and the improbable total mediation of the effects of GAEA on AD should be treated with some skepticism and await replication. Furthermore, we would caution against viewing any of our results as clinically actionable. We need a much better understanding about how high GAEA might protect against a range of psychiatric disorders before we can consider translational efforts.

## Limitations

These results should be considered in light of seven potentially significant methodological limitations. First, all of the disorders we examined, with the exception of DUD and AUD that also used criminal registries, relied solely on information obtained from medical contacts in the Swedish system. This means that affected individuals who never sought care for their condition and were not diagnosed during other medical contacts, would be missed in our study.

Second, the value of our results is dependent upon the validity of the diagnoses obtained from the Swedish registries which has been well supported generally [32] and specifically for SZ and BD [33–35]. The validity of the diagnosis of MD and AD are supported by their prevalence, sex ratio, sibling and twin correlations and associations with known psychosocial risk factors [36, 37]. The validity of our definitions of AUD and DUD is supported by the high rates of concordance across ascertainment methods [38, 39] and patterns of resemblance in relatives similar to those found in personally interviewed samples [40, 41]. However, our prevalence rates are considerably lower than in personally interviewed samples from the US [42, 43], suggesting that we are sampling from the more severe end of the substance use disorder spectrum. The diagnosis of ADHD in Sweden is validated by its close relationship with the receipt of stimulant medication [44]. We know of no specific validation of AN and BN Swedish registry diagnoses although they have been used in a number of prior research studies (e.g. [45–47]).

Third, given the differences seen in our analyses of the joint effects of genetic risk and GAEA across sexes, we wanted to examine further the stability of our findings. Therefore, we compared results for our key model c in Table 2 for 8 of our disorders across distinct historical cohorts (all but AN and BN, rare in our earlier cohort): 1973–1983 and 1984–1995 (appendix Table 5 and Fig. 1a–h). The patterns are reassuringly similar in form across both cohorts, although the shape of the OCD analyses differs from that emerging from the total sample. These results raise legitimate questions about the stability of our OCD results for the entire sample.

Fourth, the parents of children with high GAEA tend to be well educated and in a higher-than-average social class. To assess whether this might bias our studies, we repeated all the analyses depicted in Table 2 controlling for parental EA and neighborhood deprivation. As seen in appendix Table 6, these results differed very modestly from those presented here, consistent with the results of Belsky et al. where controlling for parental EA had little impact on the effect of GAEA on life course achievements [16].

Fifth, the FGRS, a family phenotype-based measure of genetic risk distinct from polygenic risk scores (PRS), has been now widely published [15, 48–53], and shown to be insensitive to assumptions involved in its calculation, with cohabitation effects performing appropriately, and agrees well with other similar statistical approaches [54, 55]. Furthermore, recent empirical analyses and simulations demonstrate that the observed modest correlations between FRGS-like statistics and PRS from the iPsych study for psychiatric disorders are consistent with the hypothesis that FGRS and PRS are both fallible measures of the same underlying genetic liability [55].

Sixth, we did not examine the impact of the likely widespread comorbidities between the psychiatric and substance use disorders on their interactions with GAEA [56, 57]. We have previously explored the etiology of comorbidities between ten pairs of these disorders in the Swedish population from a genetic-epidemiological perspective [57]. The most common pattern is for comorbid cases to have higher genetic risks across multiple disorders than those seen in individuals affected with just one of the disorders. These findings suggest that the interaction effects we have observed in these analyses with GAEA would be qualitatively similar but like quantitatively stronger in comorbid cases. Further analyses would be required to confirm this.

Lastly, the correlation observed between the genetic risk for our psychiatric disorders and GAEA may have causal elements that have not been accounted for in our analyses. For example, early onset substance use disorders or major depression might directly impair educational attainment, although we here note that a prior mendelian randomization study suggested a causal effect of genetic risk for AUD on educational attainment [58]. These effects, which will be quite complex to disentangle in both our probands and their relatives, could lead to upward biases on the association between our FGRS and GAEA measures.

## Conclusions

The impact of GAEA on risk for psychiatric and substance can be substantial and varies widely across specific disorders in magnitude, direction, and patterns of mediation. GAEA also often interacts, sometimes robustly, with disorder specific genetic risk factors. Comprehensive genetically informed risk models for psychiatric disorders should consider the inclusion of GAEA or more widely available proxies such as familial educational level.

## Supplementary information

Supplemental Material

## Data Availability

The data for this study are not publicly available due to legal restrictions with regard to the nationwide Swedish registers, but they can be acquired directly from the responsible authorities pending their approval.
