# Supplementary material for: The moderation of genetic risk for ten major psychiatric and substance use disorders by the genetic aptitude for educational attainment
Source: Mol Psychiatry. 2025 Apr 17;30(7):3160–7. doi: 10.1038/s41380-025-03022-z (PMC12185330; doi:10.1038/s41380-025-03022-z)
Supplement: Supplementary file 1 — Supplemental Material [file 41380_2025_3022_MOESM1_ESM.docx]

Appendix

**Table 1 - Description of Registers**

*National Patient Register*

In the 1960's the National Board of Health and Welfare started to collect information regarding in-patients at public hospitals, the National Patient Register (NPR). Initially it contained information about all patients treated in psychiatric care and approximately 16 percent of patients in somatic care. The register at that time covered six of the 26 county councils in Sweden. In 1984, the Ministry of Health and Welfare together with the Federation of County Councils decided a mandatory participation for all county councils. From 1987, NPR includes all in-patient care in Sweden. Since 2001, the register also covers outpatient doctor visits including day surgery and psychiatric care from both private and public caregivers. For more information, see https://www.socialstyrelsen.se/en/statistics-and-data/registers/register-information/the-national-patient-register/

*Primary Care Data*

We also used information from Primary Care. This is a research dataset including individual-level information on clinical diagnoses from primary health care centers. In the end of the follow-up period the registers covers almost 100% of the population. The figure below show the percentage of the entire Swedish population that resides in counties with primary care data. For more information see: Sundquist, J., Ohlsson, H., Sundquist, K., Kendler, KS. Common adult psychiatric disorders in Swedish primary care where most mental health patients are treated. BMC Psychiatry 17, 235 (2017). https://doi.org/10.1186/s12888-017-1381-4

*
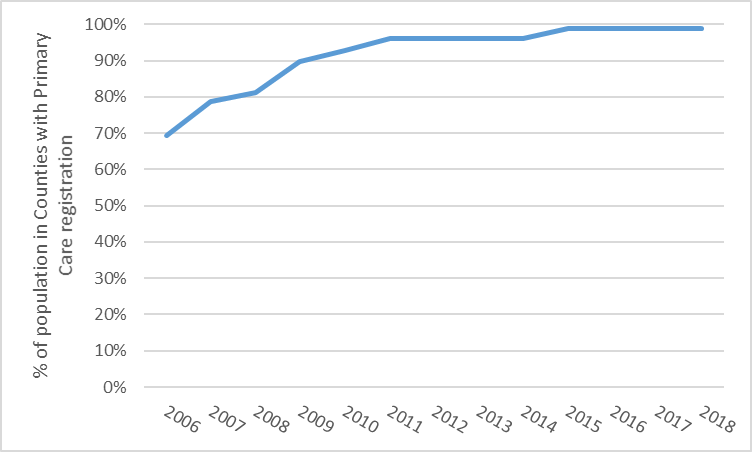
*

**Table 2 - Definition of Variables**

|  | Registers Used | Definition |
| --- | --- | --- |
| Major Depression (MD) | The National Patient Register, Primary Care data | ICD-8: 296.2, 298.0, 300.4; ICD-9: 296.2, 296.4, 298.0, 300.4; ICD-10: F32, F33. |
| Anxiety Disorder (AD) | The National Patient Register, Primary Care data | ICD-8: 300.0, 300.2 ; ICD-9: 300A, 300C; ICD-10: F40, F41 |
| Obsessive-Compulsive Disorder [OCD] | The National Patient Register, Primary Care data | ICD-9: 300D; ICD-10: F42 |
| Anorexia Nervosa (AN) | The National Patient Register, Primary Care data | ICD-9: 307B; ICD-10: F500 |
| Bulimia (BN) | The National Patient Register, Primary Care data | ICD-10: F502, F503 |
| Bipolar Disorder (BD) | The National Patient Register, Primary Care data | ICD-8: 296.1, 296.3, 296.8, 296.9, 298.1; ICD-9: 296A, 296C, 296D, 296E, 296W, 298B; ICD-10: F30, F31 |
| Schizophrenia (SZ) | The National Patient Register, Primary Care data | ICD-8: 295.1, 295.2, 2953, 295.9, 295.6; ICD-9: 295B, 295C, 295D, 295G, 295X; ICD-10: F200, F201, F202, F203, F205, F209 |
| ADHD | The National Patient Register, Primary Care data | ICD-9: 314; ICD-10: F90 |
| Alcohol Use Disorder (AUD) | The National Patient Register, Primary Care data, the Swedish Drug Register (2005-2018); the Swedish Mortality Register, and the Swedish Criminal Register (1973-2018) and the Swedish Suspicion Register (1998-2018) | Alcohol Use Disorder (AUD) was identified in the Swedish medical and mortality registries by ICD codes: ICD9: V79B, 305A, 357F, 571A-D, 425F, 535D, 291, 303, 980; ICD 10: E244, G312, G621, G721, I426, K292, K70, K852, K860, O354, T51, F10); in the Swedish Criminal Register and the Swedish Suspicion Register with at least two registrations of drunk driving (suspicion code 3005, law 1951:649 (paragraph 4 and 4A)) or drunk in charge of a maritime vessel (suspicion code 3201, law 1994:1009 (chapter 20, paragraph 4 and 5)); in the Prescribed Drug Register by the drugs disulfiram (Anatomical Therapeutic Chemical (ATC) Classification System N07BB01), acamprosate (N07BB03), and naltrexone (N07BB04). |
| Drug Use Disorder (DUD)) | The National Patient Register, Primary Care data, the Swedish Drug Register (2005-2018); the Swedish Mortality Register, and the Swedish Criminal Register (1973-2018) and the Swedish Suspicion Register (1998-2018) | Drug abuse (DA) was identified in the Swedish medical and mortality registries by ICD codes (ICD8: Drug dependence (304); ICD9: Drug psychoses (292) and Drug dependence (304); ICD10: Mental and behavioral disorders due to psychoactive substance use (F10-F19), except those due to alcohol (F10) or tobacco (F17)); in the Suspicion Register by codes 3070, 5010, 5011, and 5012, that reflect crimes related to DA; and in the Crime Register by references to laws covering narcotics (law 1968:64, paragraph 1, point 6) and drug-related driving offences (law 1951:649, paragraph 4, subsection 2 and paragraph 4A, subsection 2). DA was identified in individuals (excluding those suffering from cancer) in the Prescribed Drug Register who had retrieved (in average) more than four defined daily doses a day for 12 months from either of Hypnotics and Sedatives (Anatomical Therapeutic Chemical (ATC) Classification System N05C and N05BA) or Opioids (ATC: N02A). |
|  |  |  |
| GAEA | The National School Registry, Multigenerational Register, LISA database | For all individuals in our sample we used the Multigenerational Register to identify all 1^st^ to 5^th^ degree relatives. For these relatives, we used information on Average grade point at age 18/19, Average grade point at age 16 and Number of years of education.  Number of years of education are measured in 7 different levels  1 Pre-high school < 9 years  2 High School 9 years  3 Upper Secondary School < 3 years  4 Upper Secondary School 3 years  5 Post-secondary education < 3years  6 Post-secondary education 3 years or more  7 Research education (PhD).  All three educational variables are standardized with mean 0 and SD 1. For all relatives we took the mean Z-score for future calculations. For parents and siblings we corrected for cohabitation effects. To estimate the cohabitation effect (i.e. “shared environment”), we created a database with all individuals in the Swedish population born in Sweden 1955-1990. We also included the number of years, during ages 0-15, that individuals resided in the same household as their biological father. We thereby were able to define two kinds of families i) “not-lived-with” father families (offspring never resided for more than 1 year in the same household or in the same community as their biological father); ii) “lived-with” father (offspring resided a minimum of 13 year in the same household as their biological father. We performed a linear regression model with the Z-score for education trait in offspring as outcome and the Z-score for education in father, type of father, and their interaction as predictors. We used the interaction term as the difference of effect between genes only and genes + environment. The same approach was performed for half-siblings where we compared those who were reared together versus reared apart. (For parents this component was 0.87 and for siblings it was 0.76)  For each relative we then calculated the product using the three components: mean Z-score, cohabitation effects, proportion of shared genetic effects (0.003125 -0.5) with the proband. Then we average this product across all relatives to a proband. Then we corrected for the number of relatives. We multiplied the results from the previous step with a shrinkage factor. (Shrinkage factor (SF): B/(B+A/C). It produces more shrinkage if B and C are small and A is large. (A) = the variance of the z-score of the disorder across all relatives, (B) = the variance in the mean z-score across all probands,(C) = the weighted number of relatives for each proband. We standardized the risk score by year of birth and county of the proband into a z-score with mean 0 and SD 1. This was then used as the GAEA in the analyses. |

**Table 3 - Calculation of the Familial Genetic Risk Score (FGRS)**

|  |
| --- |
| The dataset for the calculations includes:  Column1 = Identification number of the proband (Born 1932-1995)  Column2 = Identification number of the relative (1st to 5th degree relatives)  Column3 = Proportion of shared additive genetic effects (0.03125 to 0.50) with the proband  Column4 = Year of Birth of relative  Column5 = Sex of relative  Column6 = Age at registration for trait  Column7 = Age at end of follow-up (2018-12-31 or age at death, or age at emigration whichever came first) |
| Step 1: Using all unique relatives with a registration for the disorder, we non-parametrically estimated the distribution of Age at first registration. The empirical distribution is used to obtain weights for relatives without a registration for the disorder, in order to account for the proportion of the time-at-risk period they had completed at the end of follow-up. For example, for relatives at age x at end of follow-up, the weight corresponds to the proportion of relatives registered for the trait that had been registration at age x. For relatives born prior to 1958 we subtracted age at the end of follow-up with the following formula: 1958 - Year of birth of relative. This modification was done in order to control for registration effects (i.e, most registers in Sweden start in 1973 suggesting that relatives from early birth cohorts do not have the possibility to be registered at younger ages). Note that all relatives with the disorder are weighted one. |
| Step 2: Transform the binary variable (trait yes/no) into a z-score based on the threshold for each trait. The underlying liability of the individual is not assessable. Instead we estimated the mean of the underlying liability to obtain sex and birth decade specific Z-scores for relatives with the trait registration and relatives without the trait. We generate n random numbers from a N(0, 1) distribution and estimate the mean for relatives registered with the disorder (i.e., mean of the observations above the threshold) and for relatives without a registration (i.e., mean of all observation below the threshold). The thresholds are calculated for each decade of birth and sex. |
| Step 3: Correct for cohabitation effects. To estimate the cohabitation effect (i.e. “shared environment”), we created a database with all individuals in the Swedish population born in Sweden 1955-1990. We also included the number of years, during ages 0-15, that individuals resided in the same household as their biological father. We thereby were able to define two kinds of families i) “not-lived-with” father families (offspring never resided for more than 1 year in the same household or in the same community as their biological father); ii) “lived-with” father (offspring resided a minimum of 13 year in the same household as their biological father. We performed a logistic regression model with the binary trait in offspring as outcome and the binary trait in father, type of father, and their interaction as predictors. We used the interaction term as the difference of effect between genes only and genes + environment. The same approach was performed for half-siblings where we compared those who were reared together versus reared apart. The following interaction terms were used in the calculations for each of our disorders:   \|  \| Parent/Children \| Siblings \| \| --- \| --- \| --- \| \| MD \| 0.90 \| 0.89 \| \| AD \| 0.87 \| 0.81 \| \| BD \| 0.67 \| 0.77 \| \| SZ \| 0.93 \| 0.84 \| \| AN \| * \| 0.87 \| \| BN \| * \| 0.88 \| \| OCD \| 0.79 \| 0.74 \| \| ADHD \| 0.42 \| 0.81 \| \| DUD \| 0.92 \| 0.52 \| \| AUD \| 0.99 \| 0.69 \| \| *No reliable estimate due to very low prevalence rates in males – we therefore used the mean among all other traits \| \| \| |
| Step 4: Calculate the product for each relative using the four components:   1. Z-score (reflecting sex and year of birth adjusted rates) 2. Weight (reflecting the proportion of risk period they had completed) 3. Cohabitation effects 4. Proportion of shared genetic effects (0.03125 – 0.5) with the proband |
| Step 5: Average the product calculated in step 4 across all relatives to a proband |
| Step 6: Correct for the number of relatives. We multiplied the results from step 5 with a shrinkage factor. Shrinkage factor (SF): B/(B+A/C). It produces more shrinkage if B and C are small and A is large.   1. the variance of the z-score of the disorder across all relatives, 2. the variance in the mean z-score across all probands, 3. the weighted number of relatives for each proband (sum of Column 3 across each proband). |
| Step 7: Correct for difference by year of birth and county differences. There are 21 counties in Sweden. For each proband we used the county they had resided in during the maximum number of years (measured from 1969 and onwards) We standardized the risk score by year of birth and county of the proband into a z-score with mean 0 and SD 1. |

**Table 5 – Results for Model C in Table 2 by Sex and Birth Cohort**

|  |  | GAEA | FGRS | Interaction |
| --- | --- | --- | --- | --- |
| MD | Females | 7.79 (7.68; 7.89) | 2.01 (1.91; 2.11) | 1.06 (0.96; 1.17) |
|  | Males | 4.66 (4.56; 4.76) | 0.79 (0.71; 0.86) | 0.62 (0.54; 0.69) |
|  | 1984-1995 | 7.48 (7.20; 7.58) | 1.58 (1.49; 1.68) | 0.98 (0.88; 1.07) |
|  | 1973-1983 | 5.10 (5.00; 5.20) | 1.22 (1.15; 1.30) | 0.78 (0.69; 0.86) |
| AD | Females | 7.99 (7.85; 8.13) | 1.60 (1.49; 1.71) | 1.07 (0.96; 1.17) |
|  | Males | 5.13 (5.03; 5.24) | 0.92 (0.66; 0.82) | 0.77 (0.69; 0.85) |
|  | 1984-1995 | 8.23 (8.09; 8.36) | 1.34 (1.24; 1.45) | 1.14 (1.03; 1.25) |
|  | 1973-1983 | 5.17 (5.07; 5.27) | 1.06 (0.98; 1.14) | 0.83 (0.75; 0.91) |
| OCD | Females | 0.46 (0.42; 0.50) | -0.05 (-0.07; -0.02) | 0.05 (0.01; 0.08) |
|  | Males | 0.32 (0.29; 0.36) | -0.11 (-0.13; -0.09) | 0.00 (-0.02; 0.03) NS |
|  | 1984-1995 | 0.50 (0.45; 0.54) | -0.09 (-0.12; -0.06) | 0.05 (0.01; 0.09) |
|  | 1973-1983 | 0.28 (0.24; 0.31) | -0.05 (-0.07; -0.03) | 0.01 (-0.02; 0.04) NS |
| DUD | Females | 2.12 (2.06; 2.19) | 0.84 (0.81; 0.88) | 0.68 (0.62; 0.74) |
|  | Males | 4.59 (4.49; 4.69) | 1.67 (1.62; 1.73) | 1.28 (1.20; 1.37) |
|  | 1984-1995 | 4.59 (4.49; 4.69) | 1.65 (1.59; 1.71) | 1.31 (1.22; 1.40) |
|  | 1973-1983 | 2.23 (2.17; 2.30) | 0.92 (0.88; 0.96) | 0.73 (0.67; 0.80) |
| AUD | Females | 1.33 (1.29; 1.38) | 0.51 (0.48; 0.54) | 0.42 (0.37; 0.47) |
|  | Males | 2.54 (2.48; 2.61) | 1.32 (1.27; 1.36) | 0.95 (0.88; 1.01) |
|  | 1984-1995 | 1.98 (1.92; 2.04) | 0.87 (0.83; 0.91) | 0.63 (0.57; 0.68) |
|  | 1973-1983 | 1.92 (1.87; 1.98) | 0.95 (0.91; 0.99) | 0.72 (0.66; 0.77) |
| ADHD | Females | 2.41 (2.35; 2.48) | 0.39 (0.36; 0.43) | 0.27 (0.21, 0.33) |
|  | Males | 2.87 (2.79; 2.94) | 0.74 (0.70; 0.74) | 0.46 (0.39; 0.52) |
|  | 1984-1995 | 3.84 (3.74; 3.94) | 0.99 (0.94; 1.03) | 0.65 (0.57; 0.73) |
|  | 1973-1983 | 1.60 (1.54; 1.65) | 0.22 (0.19; 0.24) | 0.24 (0.19; 0.29) |
| BD | Females | 1.06 (1.01; 1.12) | 0.18 (0.15; 0.21) | 0.11 (0.07; 0.16) |
|  | Males | 0.65 (0.61; 0.69) | -0.01 (-0.04; 0.00) | 0.01 (-0.03; 0.04) NS |
|  | 1984-1995 | 1.00 (0.95; 1.05) | 0.08 (0.06; 0.11) | 0.07 (0.03; 0.12) |
|  | 1973-1983 | 0.72 (0.68; 0.76) | 0.08 (0.06; 0.10) | 0.05 (0.02; 0.09) |
| SZ | Females | 0.10 (0.08; 0.12) | 0.00 (-0.01; 0.01) | 0.01 (0.00; 0.03) NS |
|  | Males | 0.16 (0.13; 0.18) | -0.01 (-0.03; 0.00) | 0.02 (0.00; 0.04) NS |
|  | 1984-1995 | 0.08 (0.06; 0.10) | 0.00 (0.00; 0.00) | 0.02 (0.00; 0.03) NS |
|  | 1973-1983 | 0.18 (0.16; 0.21) | -0.01 (-0.02; 0.00) | 0.02 (0.00; 0.04) NS |

**Figure 1a Major Depression**


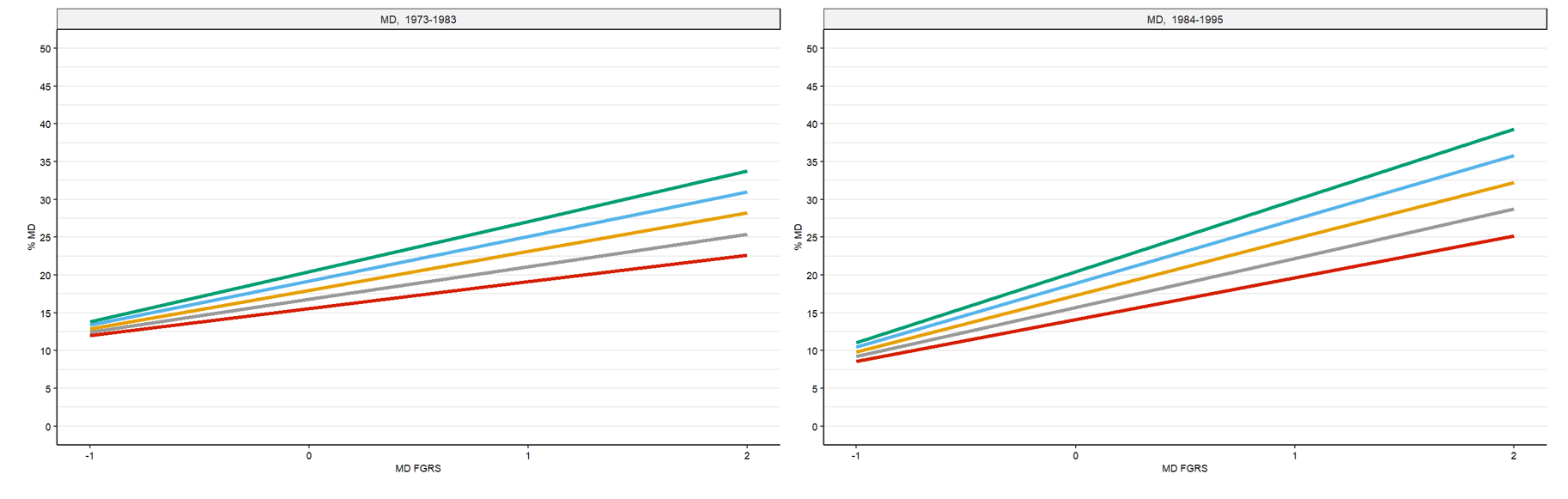


**Figure 1b – Anxiety Disorders**


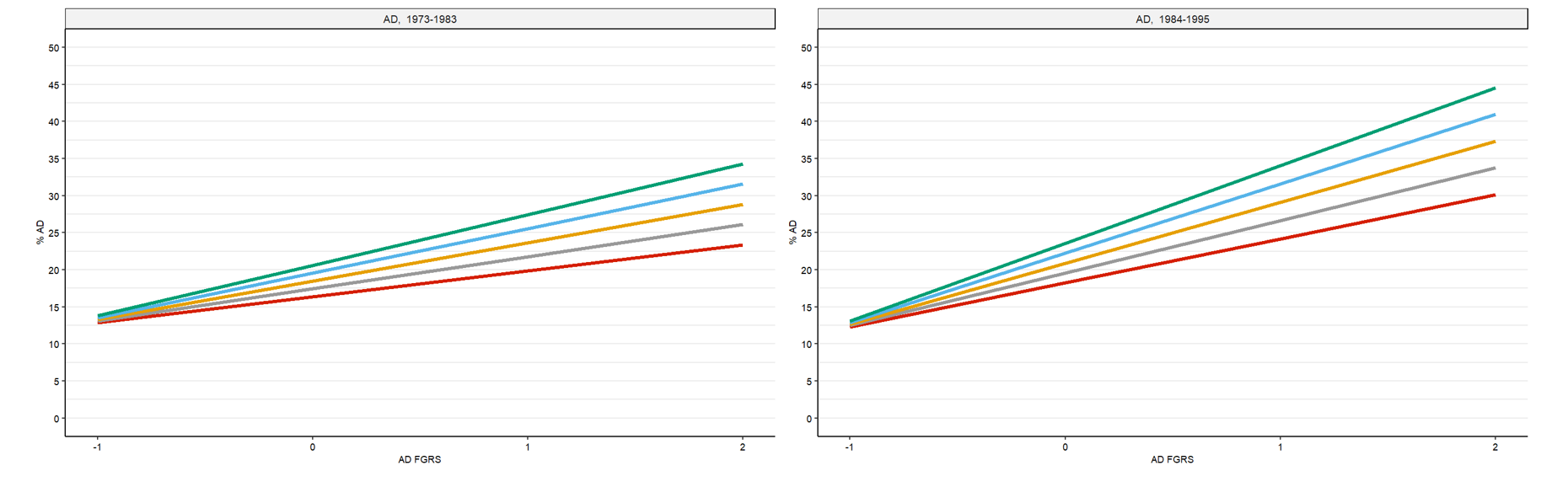


**Figure 1c - OCD**


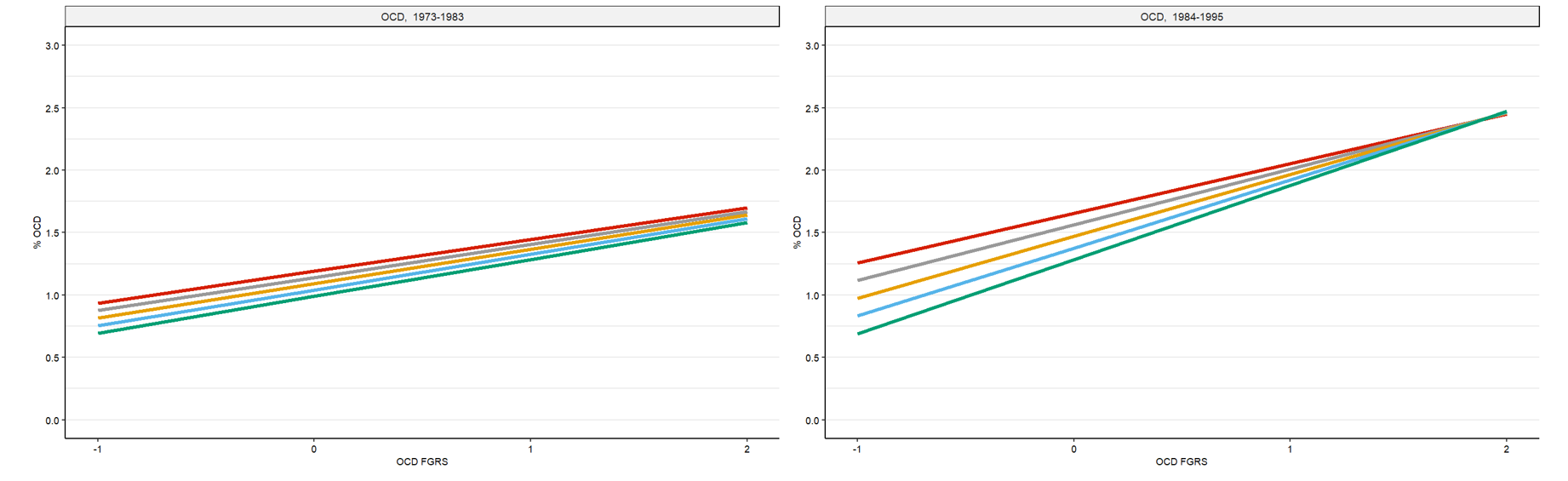


**Figure 1d Drug Use Disorder**


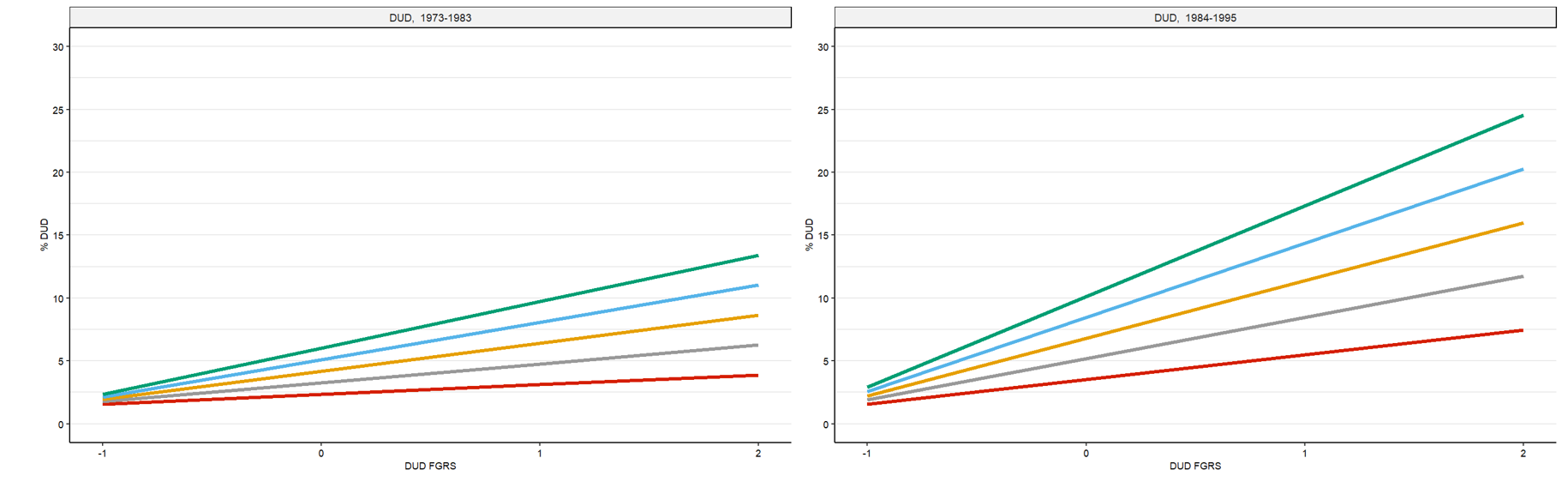


**Figure 1e Alcohol Use Disorder**


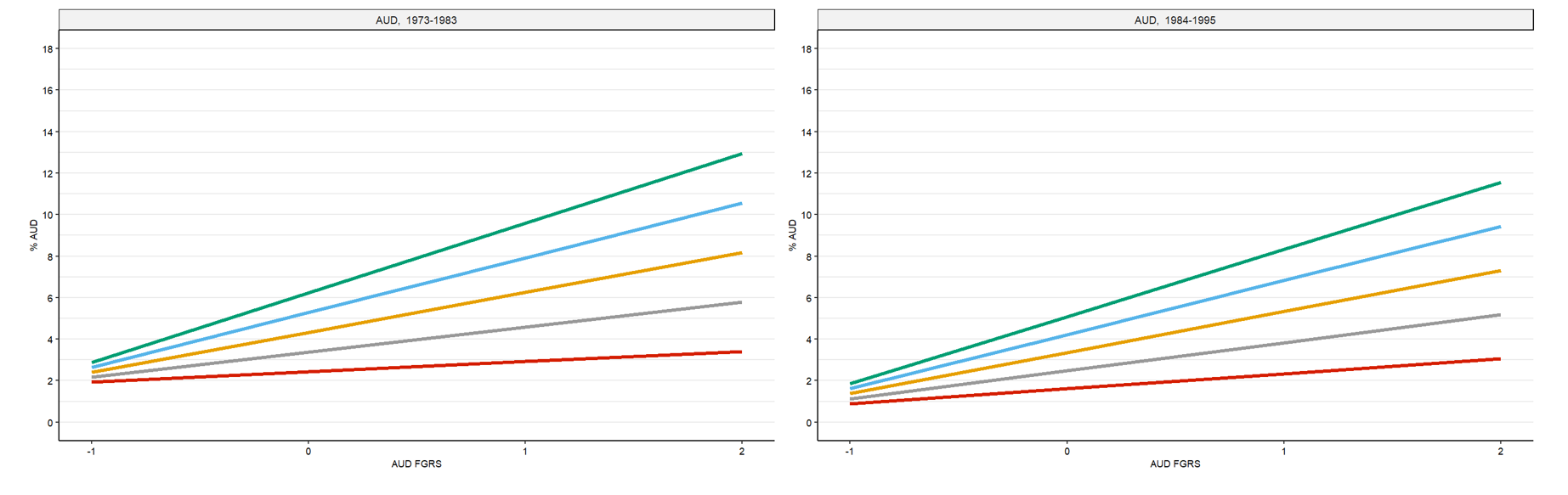


**Figure 1f ADHD**


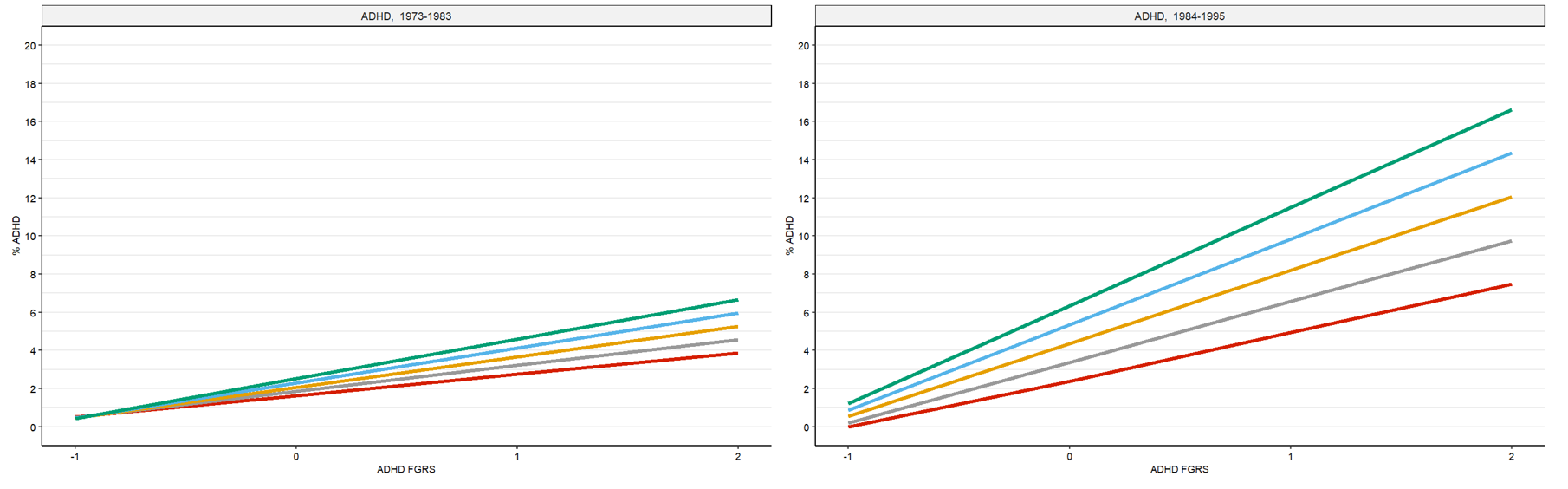


**Figure 1g Bipolar Disorder**


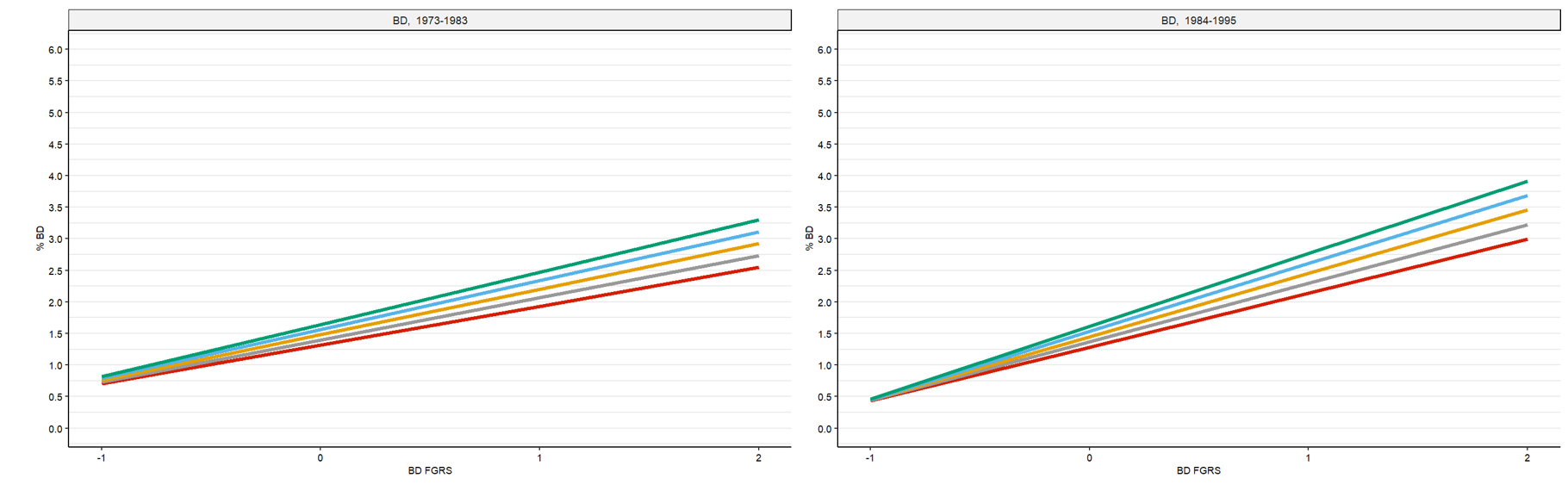


**Figure 1h Schizophrenia**


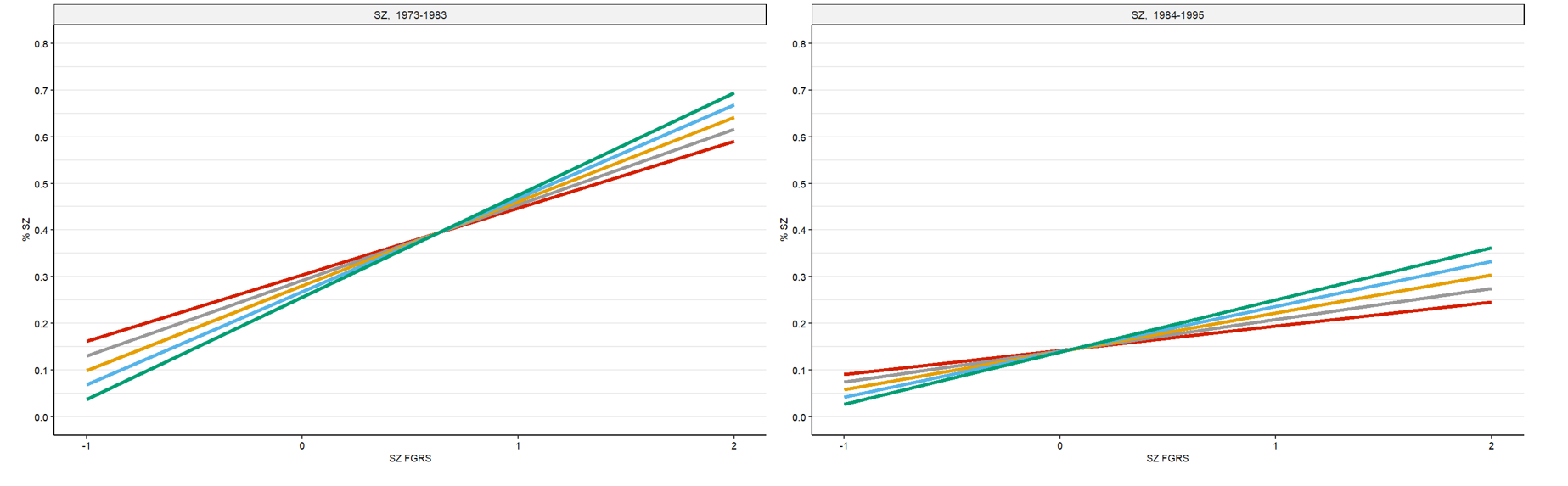


| Table 6 - Results from Aalens Linear Hazard models which adds – from table 2 in the main manuscripts – models A2, B2 and C2 which add to the model models controls for Parental Income at age 17 and Neighborhood deprivation at age 17. | | | | | | | |
| --- | --- | --- | --- | --- | --- | --- | --- |
|  |  | Model A | Model A2 | Model B | Model B2 | Model C | Model C2 |
| DUD | DUD FGRS | 3.98 (3.91; 4.05) | 3.84 (3.74; 3.91) | 3.71 (3.64; 3.74) | 3.67 (3.60; 3.74) | 3.35 (3.29; 3.40) | 3.32 (3.26; 3.37) |
|  | EDU FGRS | 1.92 (1.89; 1.96) | 1.65 (1.62; 1.68) | 1.15 (1.12; 1.18) | 0.95 (0.91; 0.98) | 1.25 (1.22; 1.29) | 1.06 (1.02; 1.09) |
|  | Interaction |  |  |  |  | 0.96 (0.90; 1.01) | 0.95 (0.90; 1.00) |
|  |  |  |  |  |  |  |  |
| AUD | AUD FGRS | 2.33 (2.29; 2.37) | 2.26 (2.22; 2.30) | 2.33 (2.29; 2.37) | 2.11 (2.07; 2.16) | 1.95 (1.91; 1.99) | 1.93 (1.89; 1.97) |
|  | EDU FGRS | 1.32 (1.29; 1.35) | 1.17 (1.15; 1.20) | 0.82 (0.80; 0.85) | 0.72 (0.69; 0.75) | 0.91 (0.89; 0.94) | 0.82 (0.79; 0.85) |
|  | Interaction |  |  |  |  | 0.68 (0.64; 0.71) | 0.67 (0.64; 0.71) |
|  |  |  |  |  |  |  |  |
| ADHD | ADHD FGRS | 2.87 (2.82; 2.92) | 2.83 (2.78; 2.88) | 2.76 (2.71; 2.82) | 2.75 (2.70; 2.81) | 2.63 (2.58; 2.68) | 2.62 (2.58; 2.81) |
|  | EDU FGRS | 1.09 (1.06; 1.12) | 0.96 (0.94; 0.99) | 0.55 (0.53; 0.58) | 0.46 (0.43; 0.48) | 0.57 (0.54; 0.60) | 0.47 (0.44; 0.48) |
|  | Interaction |  |  |  |  | 0.35 (0.31; 0.39) | 0.35 (0.31; 0.39) |
|  |  |  |  |  |  |  |  |
| MD | MD FGRS | 6.19 (6.12; 6.26) | 6.09 (6.02; 6.15) | 6.09 (6.02; 6.15) | 6.02 (5.95; 6.09) | 6.15 (6.09; 6.22) | 6.09 (6.02; 6.15) |
|  | EDU FGRS | 1.88 (1.82; 1.94) | 1.88 (1.82; 1.94) | 1.42 (1.36; 1.48) | 1.04 (0.98; 1.11) | 1.38 (1.32; 1.44) | 0.99 (0.92; 1.05) |
|  | Interaction |  |  |  |  | 0.84 (0.78; 0.90) | 0.85 (0.79; 0.91) |
|  |  |  |  |  |  |  |  |
| AD | AD FGRS | 6.63 (6.56; 6.70) | 6.53 (6.46; 6.60) | 6.43 (6.36; 6.49) | 6.43 (6.36; 6.49) | 6.46 (6.39; 6.53) | 6.43 (6.36; 6.49) |
|  | EDU FGRS | 1.99 (1.92; 2.05) | 1.53 (1.47; 1.60) | 0.81 (0.73; 0.88) | 0.78 (0.71; 0.85) | 1.15 (1.09; 1.22) | 0.78 (0.71; 0.85) |
|  | Interaction |  |  |  |  | 0.91 (0.84; 0.98) | 0.91 (0.85; 0.98) |
|  |  |  |  |  |  |  |  |
| AN | AN FGRS | 0.09 (0.08; 0.11) | 0.09 (0.08; 0.11) | 0.09 (0.08; 0.11) | 0.09 (0.08; 0.11) | 0.09 (0.07; 0.10) | 0.09 (0.07; 0.10) |
|  | EDU FGRS | -0.13 (-0.15; -0.12) | -0.13 (-0.14; -0.12) | -0.13 (-0.15; -0.12) | -0.13 (-0.14; -0.12) | -0.14 (-0.15; -0.12) | -0.13 (-0.14; -0.12) |
|  | Interaction |  |  |  |  | -0.02 (-0.04; -0.01) | -0.02 (-0.04; -0.01) |
|  |  |  |  |  |  |  |  |
| BN | BN FGRS | 0.04 (0.03; 0.05) | 0.04 (0.03; 0.05) | 0.04 (0.03; 0.05) | 0.04 (0.03; 0.05) | 0.04 (0.03; 0.05) | 0.04 (0.03; 0.05) |
|  | EDU FGRS | -0.06 (-0.07; -0.06) | -0.06 (-0.07; -0.05) | -0.06 (-0.07; -0.05) | -0.06 (-0.07; -0.05) | -0.06 (-0.15; -0.12) | -0.06 (-0.07; -0.05) |
|  | Interaction |  |  |  |  | -0.01 (-0.02; 0.00)NS | -0.01 (-0.02; 0.00)NS |
|  |  |  |  |  |  |  |  |
| OCD | OCD FGRS | 0.38 (0.26; 0.41) | 0.39 (0.36; 0.41) | 0.38 (0.36; 0.41) | 0.38 (0.36; 0.41) | 0.39 (0.36; 0.41) | 0.39 (0.36; 0.41) |
|  | EDU FGRS | -0.08 (-0.09; -0.06) | -0.10 (-0.12; -0.08) | -0.08 (-0.10; -0.06) | -0.09 (-0.11; -0.07) | -0.08 (-0.10; -0.06) | -0.10 (-0.12; -0.08) |
|  | Interaction |  |  |  |  | 0.02 (0.00; 0.05) | 0.02 (0.00; 0.05) |
|  |  |  |  |  |  |  |  |
| BD | BD FGRS | 0.08 (0.08; 0.09) | 0.08 (0.08; 0.09) | 0.08 (0.08; 0.09) | 0.08 (0.08; 0.09) | 0.09 (0.08; 0.09) | 0.08 (0.08; 0.09) |
|  | EDU FGRS | 0.09 (0.07; 0.10) | 0.03 (0.01; 0.05) | 0.08 (0.07; 0.10) | 0.04 (0.02; 0.05) | 0.08 (0.06; 0.10) | 0.03 (0.01; 0.05) |
|  | Interaction |  |  |  |  | 0.06 (0.03; 0.09) | 0.06 (0.03; 0.09) |
|  |  |  |  |  |  |  |  |
| SZ | SZ FGRS | 0.13 (0.12; 0.15) | 0.13 (0.12; 0.15) | 0.13 (0.12; 0.15) | 0.13 (0.12; 0.15) | 0.13 (0.12; 0.15) | 0.13 (0.12; 0.15) |
|  | EDU FGRS | -0.00 (-0.01; 0.00) | -0.01 (-0.02; -0.01) | -0.01 (-0.01; 0.00) | -0.01 (-0.02; 0.00) | -0.01 (-0.01; 0.00) | -0.02 (-0.02; 0.00) |
|  | Interaction |  |  |  |  | 0.02 (0.00; 0.03) | 0.02 (0.00; 0.03) |
